# Effect of initial switch-on within 24 hours of cochlear implantation using slim modiolar electrodes

**DOI:** 10.1038/s41598-021-01862-7

**Published:** 2021-11-23

**Authors:** Woongsang Sunwoo, Hyoung Won Jeon, Byung Yoon Choi

**Affiliations:** 1 Department of Otorhinolaryngology-Head and Neck Surgery, Gil Medical Center, Gachon University College of Medicine, Incheon, Republic of Korea; 2 Department of Otorhinolaryngology-Head and Neck Surgery, Seoul National University Bundang Hospital, Seoul National University College of Medicine, 300 Gumi-dong, Bundang-gu, Seongnam, 13620 Republic of Korea

**Keywords:** Medical research, Auditory system

## Abstract

Reducing electrode impedance is an important factor in improving the functional benefits of cochlear implants (CIs). The immediate effect of early switch-on within 24 h of surgery on impedance among CI recipients with various types of electrodes has been reported previously; however, the immediate change and the evolution of electrode impedances of slim modiolar electrodes after early switch-on within 24 h of implantation has not. Therefore, the focus of this retrospective cohort study of CI patients was to compare the effect of early switch-on (n = 36) and conventional switch-on (n = 72) 2–4 weeks post-operation on impedance. Compared with impedance measured intraoperatively, our results demonstrate a significant decrease in impedance from 11.5 to 8.9 kΩ (*p* < 0.001) at 2–4 weeks after implantation in the early switch-on group, which sharply contrasted with elevated impedance values for conventional switch-on 2–4 weeks after implantation (from 10.7 to 14.2 kΩ, *p* = 0.001). Notably, a comparatively lower impedance than the conventional switch-on protocol was observed for up to 2 months post-operation. Most importantly, a much earlier stabilization of impedance can be achieved with the early switch-on protocol coupled with the slim modiolar electrode array compared to the conventional switch-on protocol, offering the advantage of reducing the number of required mapping sessions in the early stages of rehabilitation.

## Introduction

In most cochlear implant (CI) centers currently, electrode impedance measurements are routinely conducted immediately after electrode insertion during CI and at the beginning of every programming session^1,2^. Software-based impedance telemetry has evolved to assist in the objective assessment of internal implant device functionality. Although the internal CI device reliability is high, previous studies have determined that electrode failures can occur in up to 7–9% of recipients^3–5^. In addition to detecting electrode abnormalities, monitoring electrode impedance provides guidance for subsequent setting of stimulation parameters of the device. Electrode impedance is influenced by the physical properties of the electrode contacts as well as the medium surrounding the electrode, such as macrophages, proteins, fibrous tissues, and perilymph^6,7^. Thus, intracochlear changes due to insertional trauma or an immunologic response to the electrode cause postoperative increases in impedance^8,9^.

The use of an electrode with a high impedance value may compromise sound quality, speech perception, and the loudness perception^10^. Higher impedances require the generation of higher voltages across the electrode-tissue interface to achieve a satisfactory level of loudness. Owing to the compliance voltage limit of the CI device, the maximum current that can be delivered to the electrode contacts with high impedance values is necessarily reduced, which in turn decreases the dynamic range of the current stimulation. Therefore, reducing electrode impedance is an important aspect of CI surgery and subsequent mappings to improve functional benefits.

**Figure 1:**
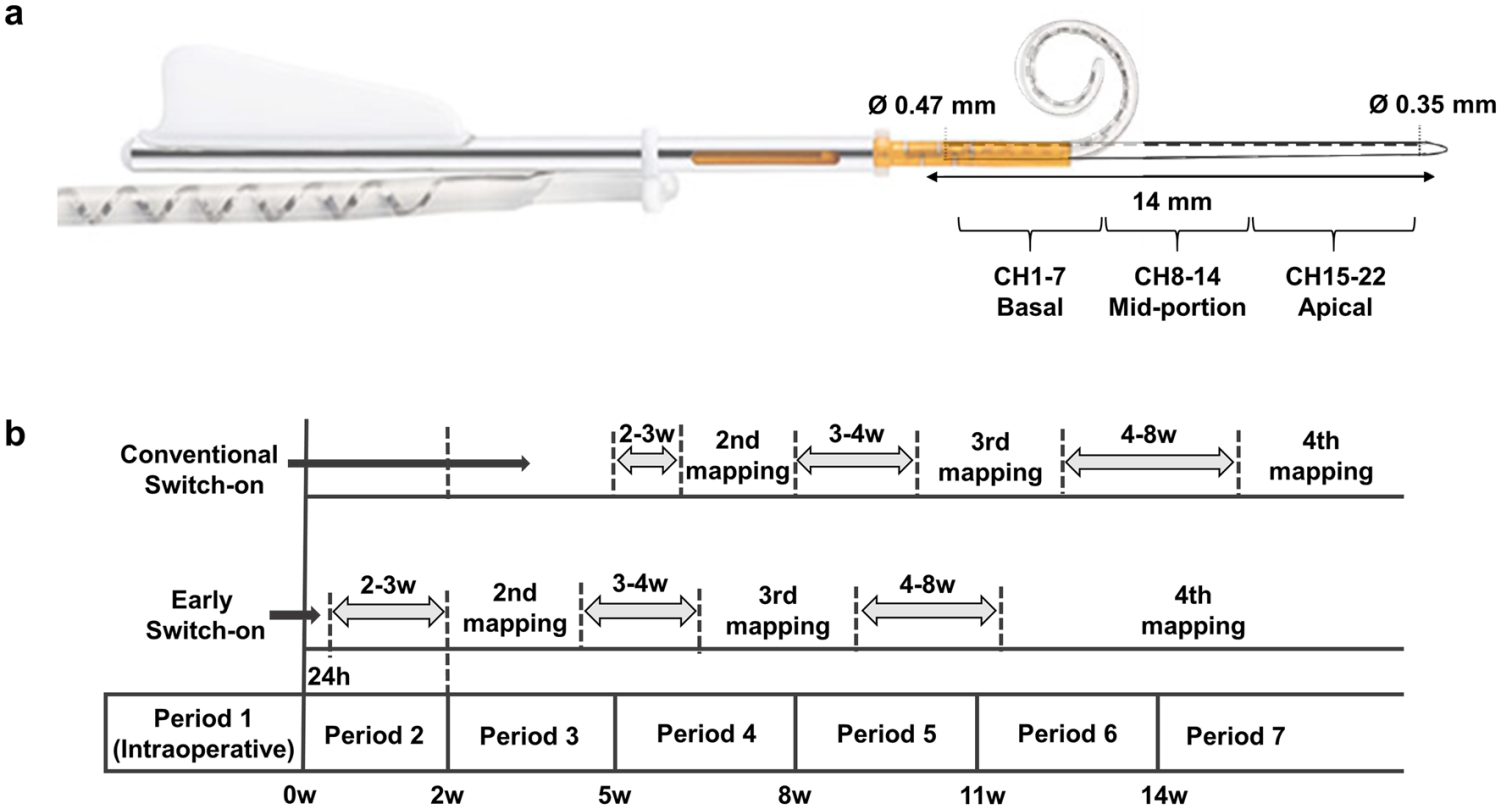
Impedance measurements with the slim modiolar electrode. (**a**) The design of the slim modiolar electrode (CI632) in sheath. 22 contacts spread over 14 mm active length is classified as the basal (CH 1–7), mid‐portion (CH 8–14), and apical (CH 15–22) segment. (**b**) Timeline for the mapping process in both early switch-on and conventional switch-on groups showing times relative to cochlear implantation (0 week) for five impedance measures: (1) intraoperative; (2) at the initial switch-on; (3) 2–3 weeks after the first mapping; (4) 3–4 weeks after the second mapping; and (5) 4–8 weeks after the third mapping.

Atraumatic insertion is an important prerequisite for minimizing the increase in electrode impedance levels, since inflammatory responses after mechanical trauma have been considered the primary cause of cochlear fibrosis and ossification^11,12^. Electrode design is also one of the major factors contributing to the damage to the cochlear structure during electrode insertion. Recently, a new type of slim modiolar electrode (CI532 and CI632, Fig. 1a) has been developed by Cochlear Ltd. (Sydney, Australia) to protect and preserve the delicate structures of the cochlea, while obtaining a tight modiolar proximity. Due to its pre-curved perimodiolar design without a stylet, injury to the basilar membrane and the lateral wall of the scala tympani can be avoided. Moreover, since its thin profile has 60% less volume than previous perimodiolar electrodes (CI512), a lower hydrodynamic load can be expected at the distal end of the cochlea^13^. Several studies have reported that insertion of the slim modiolar electrode can be less traumatic, as indicated by the higher rate of residual hearing preservation^14,15^.

Electrical stimulation can also alter electrode impedance^16^. In general, the impedance of implanted electrodes decreases temporarily after stimulation and increases during periods of non-stimulation. Therefore, it can be inferred that the timing of device activation after implantation can also affect the change in impedance. Since the efficacy and safety of early switch-on within 24 h of surgery has previously been confirmed^17^, many CI centers, including ours, have changed the switch-on protocol over the past few years from the initial fitting at 3–6 weeks post-surgery to the day after surgery. Previous studies investigating the evolution of electrode impedance after early switch-on have shown inconsistent results according to the type of electrode array^18–20^. To the best of our knowledge, this is the first study to describe changes in the impedance of slim modiolar electrodes over time in CI recipients who were rehabilitated with the conventional and early switch-on protocols. The aim of this study was to determine the changes in electrode impedance over time in a relatively large group of participants who were implanted with the slim modiolar electrode for up to 3–4 months after surgery. We also investigated whether the evolution of electrode impedances systematically varied between the two different switch-on protocols and assessed the effect of early initiation of stimulation on impedance changes.

## Methods

### Ethical statement

This study was approved by the institutional review board (IRB) of Seoul National University Bundang Hospital (No. B-2008/630-108). The IRB also approved a waiver of informed consent for this retrospective chart review, which involves no more than minimal risk. All methods carried out in this study were in accordance with the approved guidelines, regulations, and the Declaration of Helsinki.

### Subjects

84 patients (108 ears) who underwent cochlear implantation by a single surgeon (BY Choi) at Seoul National University Bundang Hospital from July 2018 to July 2020 were recruited for this study. All patients underwent CI exclusively with a slim modiolar electrode (CI532 or CI632) (Cochlear Ltd., Sydney, Australia). The switch-on of the device and the first mapping were performed within 24 h of cochlear implantation for 36 ears (26 subjects), and this group was designated the early switch-on group. For the other 72 ears (58 subjects), device switch-on and the first mapping were performed two to four weeks after surgery, and this group was classified as the conventional switch-on group. To be matched with the early switch-on group (n = 36 ears), 72 ears, twice the number of ears of the early switch-on group, were randomly assigned to the conventional mapping group with age and gender matching between the two groups.

Impedance was measured once during surgery (intraoperative) and was performed at least four times thereafter, once for each of the four consecutive mappings. In both groups, the second mapping was performed 2–3 weeks after the first mapping, the third mapping was performed 3–4 weeks after the second mapping, and the fourth mapping was performed 4–8 weeks after the third mapping (Fig. 1b). We defined CH1‐CH7 as basal electrodes, CH8‐CH14 as mid‐portion electrodes, and CH15‐CH22 as apical electrodes (Fig. 1a).

### Mode of impedance measurement

The impedance measurement modes include the monopolar, bipolar, and common ground (CG) modes. Among them, we measured the impedance level using both the monopolar 2 (MP2) mode, where the reference is the plate electrode, and the CG mode. Previously, impedance measurements using the CG mode provided an impedance level that was 13.3% lower than that using the MP2 mode^18^, and we intended to analyze the impedance change based on both modes to strengthen our argument. Because there were no significant differences between the two modes, here we primarily reported the impedance under the MP2 mode (Table 1), and the detailed impedance values under the CG mode were provided in supplementary Table S1.

**Table 1:** Mean impedance values of all channels of slim modiolar electrodes under MP2 mode.

	Period 1		Period 2	Period 3		Period 4		Period 5		Period 6		Period 7	
	Conventional	Early	Early	Conventional	Early	Conventional	Early	Conventional	Early	Conventional	Early	Conventional	Early
**Basal Electrodes (kΩ)**													
CH 01	10.63	11.88	4.54	12.80	7.75	10.23	8.21	9.90	8.12	9.57	8.66	9.74	8.96
CH 02	11.36	12.15	4.79	13.12	7.85	9.97	8.15	9.84	8.16	9.19	8.52	9.31	8.62
CH 03	11.83	12.18	4.87	13.46	8.44	10.17	8.44	9.95	8.72	9.15	8.85	9.33	8.77
CH 04	11.65	12.49	5.16	13.62	8.83	10.14	8.84	9.74	8.85	8.85	9.30	9.12	8.68
CH 05	11.58	12.61	5.23	13.42	8.65	10.05	8.94	9.59	8.66	8.61	8.73	8.87	8.63
CH 06	11.94	12.75	5.20	13.42	8.75	10.10	8.78	9.67	8.67	8.48	8.57	8.86	8.49
CH 07	11.70	12.24	5.23	13.15	8.78	10.08	8.83	9.65	8.66	8.36	8.93	8.81	8.32
Mean (95% CI)	11.53 (11.31–11.74)	12.33 (12.02–12.63)	5.00 (4.89–5.12)	13.28 (13.01–13.56)	8.44 (8.14–8.73)	10.11 (9.86–10.35)	8.60 (8.32–8.88)	9.76 (9.53–9.99)	8.55 (8.19–8.91)	8.89 (8.66–9.12)	8.79 (8.40–9.18)	9.15 (8.88–9.41)	8.64 (8.25–9.02)
**Mid-portion Electrodes (kΩ)**													
CH 08	10.91	11.72	5.15	12.99	8.66	9.99	8.98	9.41	8.75	8.18	8.87	8.60	8.40
CH 09	10.75	11.53	5.01	13.06	8.69	9.78	8.91	9.26	8.59	8.03	8.76	8.58	8.36
CH 10	10.56	11.27	5.03	12.99	8.82	9.70	8.88	9.28	8.60	8.00	8.30	8.50	8.27
CH 11	10.66	11.10	5.01	13.13	8.78	9.65	8.52	9.10	8.25	7.76	8.17	8.25	8.33
CH 12	10.62	10.84	5.17	13.01	8.76	9.62	8.73	8.95	8.18	7.65	8.50	8.21	8.25
CH 13	10.93	11.17	5.29	13.26	8.75	9.71	8.75	9.05	8.30	7.69	8.56	8.42	8.20
CH 14	10.91	11.65	5.44	13.47	9.18	9.89	8.81	9.21	8.67	7.81	8.64	8.55	8.84
Mean (95% CI)	10.76 (10.58–10.95)	11.33 (11.05–11.6)	5.16 (5.05–5.26)	13.13 (12.88–13.38)	8.81 (8.57–9.04)	9.76 (9.53–9.99)	8.80 (8.56–9.03)	9.18 (8.95–9.41)	8.48 (8.16–8.79)	7.88 (7.67–8.08)	8.54 (8.24–8.85)	8.44 (8.22–8.67)	8.38 (7.98–8.78)
**Apical Electrodes (kΩ)**													
CH 15	10.84	11.66	5.45	13.60	9.39	10.12	9.07	9.40	8.79	8.04	8.73	8.72	8.63
CH 16	10.39	11.34	5.36	13.62	9.48	9.99	9.24	9.31	8.69	8.10	8.99	8.57	8.53
CH 17	10.38	11.16	5.23	13.61	9.38	10.07	9.11	9.26	8.57	8.08	9.08	8.57	8.45
CH 18	10.46	11.48	5.40	13.67	9.57	10.26	9.30	9.35	8.65	8.21	8.86	8.48	8.63
CH 19	9.82	10.79	5.32	13.49	9.38	9.84	9.48	9.21	8.68	8.12	8.97	8.24	8.50
CH 20	9.52	10.47	5.32	13.36	9.31	9.96	9.29	9.24	8.27	8.00	8.60	8.54	8.17
CH 21	8.57	9.92	5.40	13.58	9.46	10.06	9.48	9.45	8.24	8.47	8.61	8.84	8.10
CH 22	9.65	10.19	5.25	13.75	9.42	10.21	8.70	9.34	8.08	8.34	8.09	8.47	7.55
Mean (95% CI)	9.95 (9.75–10.15)	10.87 (10.59–11.16)	5.34 (5.24–5.44)	13.59 (13.38–13.80)	9.42 (9.20–9.64)	10.06 (9.87–10.26)	9.21 (9.01–9.41)	9.32 (9.12–9.52)	8.50 (8.20–8.79)	8.17 (7.98–8.36)	8.74 (8.49–8.99)	8.55 (8.30–8.80)	8.32 (7.99–8.65)
**Total Electrodes (kΩ)**													
Mean (95% CI)	10.71 (10.27–11.16)	11.48 (10.85–12.11)	5.18 (4.97–5.38)	14.21 (13.82–14.60)	8.91 (8.32–9.50)	9.74 (9.23–10.27)	8.88 (8.36–9.41)	9.42 (8.87–9.96)	8.51 (7.73–9.28)	8.30 (7.82–8.79)	8.69 (7.99–9.40)	8.71 (8.10–9.32)	8.43 (7.59–9.27)

### Periods used for mapping

The early switch-on and conventional switch-on groups differed from each other according to the timing of the device switch-on. In order to effectively compare the impedance level between the groups at each mapping over the 4 months following implantation, the total follow-up period was divided into seven sections, and the impedance values in each of these sections were compared between the two groups. The seven sections were as follows: period 1, intraoperative period; period 2, < 2 weeks after surgery; period 3, 2–4 weeks after surgery; period 4, 5–7 weeks after surgery; period 5, 8–10 weeks after surgery; period 6, 11–13 weeks after surgery; and period 7, more than 14 weeks after surgery (Fig. 1b). Since all data within the period 2 correspond to the 24-h impedance measures in the early switch-on group, and which have not been measured in the conventional switch-on group, these period 2 data were not used for further analysis in this study.

### Statistical analysis

Descriptive data are expressed as median (range) or mean ± standard deviation (SD), as appropriate. The Shapiro–Wilk normality test was used to determine if a variable was well-modeled by a normal distribution in some population. For the statistical analysis, we compared the average impedance levels at the same period between the early switch-on group and the conventional switch-on group using a non-parametric test, the Mann–Whitney U test. Differences between impedance values measured at different periods were analyzed using the Skillings-Mack test with post hoc tests by running separate Wilcoxon signed-rank tests with a Bonferroni correction. Differences in impedance values between different electrode segments were tested using the Kruskal–Wallis test. A simple linear correlation analysis using Pearson’s correlation coefficient was used to assess the relationship between the time of cochlear implant device usage and changes in overall impedance value. Statistical significance was set at *p* < 0.05.

## Results

### Comparison of longitudinal changes in electrical impedance of slim modiolar electrodes depending on the timing of the initial switch-on

Analysis of impedance values measured at different periods showed a statistically significant difference in impedance of electrodes depending on the period of measurement in both groups; χ^2^(5) = 115.4, *p* < 0.001 in the early switch-on group and χ^2^(5) = 785.4, *p* < 0.001 in the conventional switch-on group. Post hoc analysis with Wilcoxon signed-rank tests was conducted with a Bonferroni correction applied, resulting in a significance level set at *p* < 0.003 (Table 2).

**Table 2:** Comparison of impedances between consecutive periods.

Group	Period 1 vs 3	Period 3 vs 4	Period 4 vs 5	Period 5 vs 6	Period 6 vs 7
Early switch-on	Z = − 4.672	Z = − 1.153	Z = − 1.125	Z = 0.000	Z = − 1.342
	*p* < 0.001*	*p* = 0.249	*p* = 0.260	*p* = 1.000	*p* = 0.180
Conventional switch-on	Z = − 7.211	Z = − 6.790	Z = − 4.107	Z = − 4.329	Z = − 1.481
	*p* < 0.001*	*p* < 0.001*	*p* < 0.001*	*p* < 0.001*	*p* = 0.139

*Statistical significance with a Bonferroni correction applied (*p* < 0.003).

**Figure 2:**
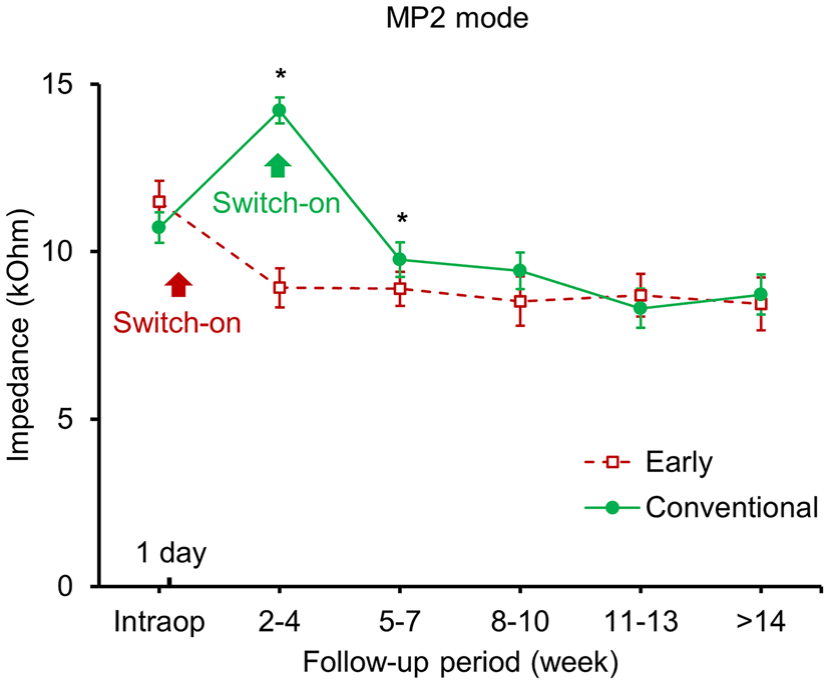
Longitudinal impedance changes of a slim modiolar electrode under monopolar 2 (MP2) mode in the early switch-on and conventional switch-on groups. Arrows indicates the period when the switch-on occurs for each group. Error bars represent ± 2 standard errors, which corresponds to a 95% confidence interval. Asterisks indicate statistically significant difference between two groups (*p* < 0.05, Mann–Whitney U test).

This longitudinal change under the MP2 mode in the early switch-on group was compared against that of the electrical impedance of the slim modiolar electrodes when the initial switch-on was made 2–4 weeks after the operation (Fig. 2). In the early switch-on group, the impedance value decreased from 11.48 ± 1.88 to 8.91 ± 1.66 kΩ until the third to fourth week after surgery. In contrast, the impedance value of the conventional switch-on group had increased by 32.7% (from 10.71 ± 1.90 to 14.2 ± 1.66 kΩ). Statistically lower impedance was observed in the early switch-on group compared with the conventional switch-on group that persisted throughout the initial 7 weeks postoperatively (*p* < 0.001 at Period 3 and *p* = 0.028 at Period 4). At 8 weeks or thereafter, there were no differences in the electrical impedance of the slim modiolar electrodes between the two groups (*p* = 0.098, *p* = 0.322, and *p* = 0.389 at Period 5, 6, and 7, respectively).

Importantly, a large difference in the timing at which the impedance stabilized between the two groups was noted. Specifically, the impedance values stabilized much earlier in the early switch-on group than in the conventional switch-on group. For the early switch-on group, the impedance value reached a plateau at period 3 (8.91 ± 1.66 kΩ; 2–4 weeks after surgery) and stabilized thereafter (Fig. 2 and Table 2). However, impedances decreased over time from the period 3 (14.21 ± 1.66 kΩ) until period 6 (8.30 ± 1.55 kΩ 13 weeks after surgery) in the conventional switch-on group. Statistically significant differences in impedance were observed for the conventional switch-on group in period 4, 5, and 6 compared to the preceding one (*p* < 0.001 for all comparison, Table 2).

**Figure 3:**
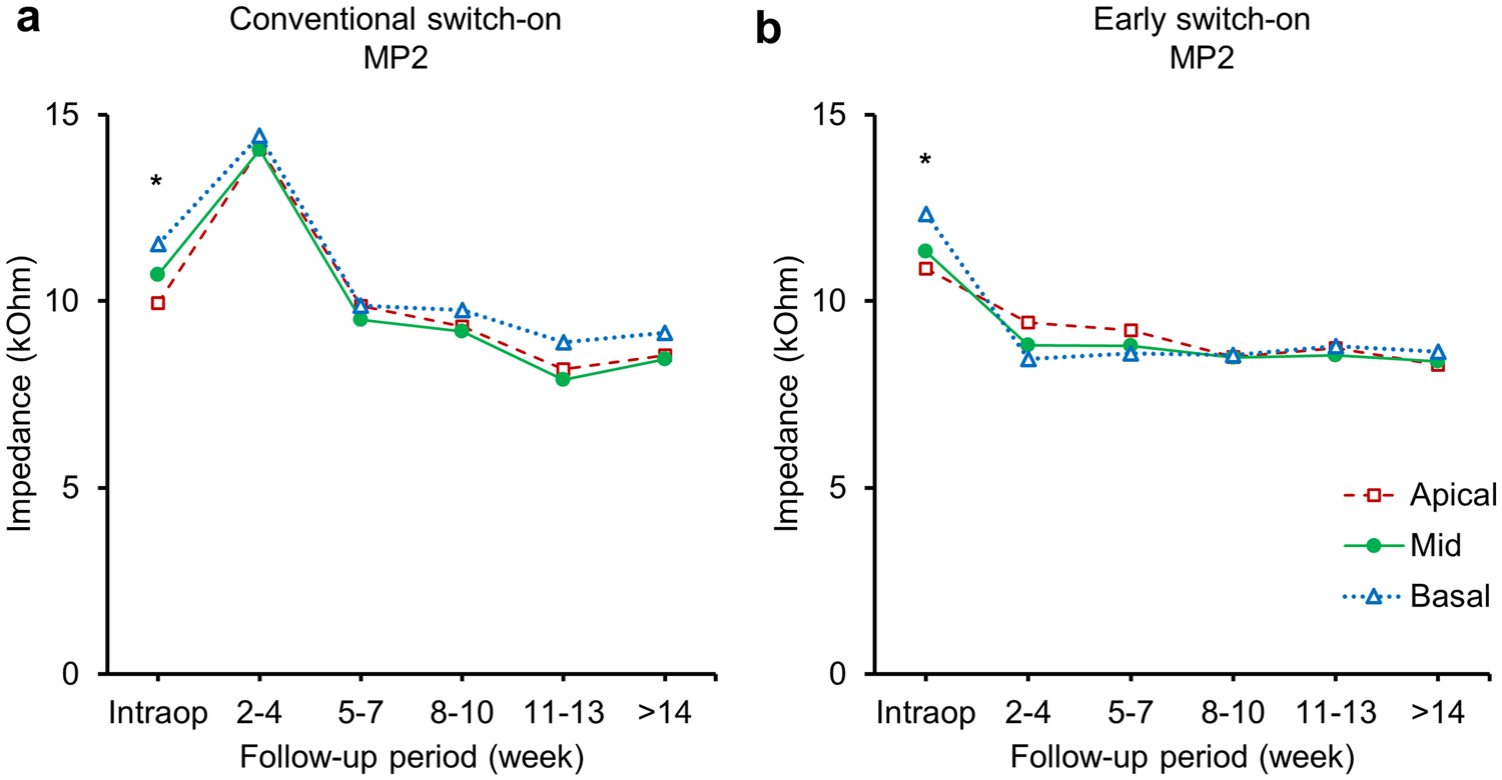
Comparison of longitudinal impedance changes according to electrode segment between early switch-on and conventional switch-on under monopolar 2 (MP2) mode (**a** and **b**). Different shapes of each data point indicate average impedances of different electrode segments. △: CH1‐CH7 as basal electrodes, ●: CH8‐CH14 as mid‐portion electrodes, □: CH15‐CH22 as apical electrodes. *p < 0.05.

Figure 3 shows the pattern of longitudinal change in electrical impedance of each electrode segments in both groups. A Kruskal–Wallis test showed that there was a statistically significant difference in impedance between the different electrode segments only at period 1, χ^2^(2) = 20.166, *p* < 0.001 for the conventional switch-on group and χ^2^(2) = 8.850, *p* = 0.012 for the early switch-on group. Post hoc analysis with Mann–Whitney U tests was conducted with a Bonferroni correction applied, resulting in a significance level set at *p* < 0.017. Electrode impedance values measured intraoperatively were significantly higher for basal electrodes compared with those for apical electrodes in both groups (*p* < 0.001 for the conventional switch-on group and *p* = 0.004 for the early switch-on group). Thereafter, the differences between apical and basal electrodes were not significant in the early switch-on group at period 3, 4, 5, 6, and 7 with *p* values of 0.062, 0.171, 0.935, 0.858, and 0.796, respectively. Similarly, non-significant differences between apical and basal electrodes were obtained in the conventional switch-on group at period 3, 4, 5, 6, and 7 with *p* values of 0.353, 0.750, 0.288, 0.071, and 0.277, respectively.

### Comparison of changes in impedance over cochlear implant device usage time

**Figure 4:**
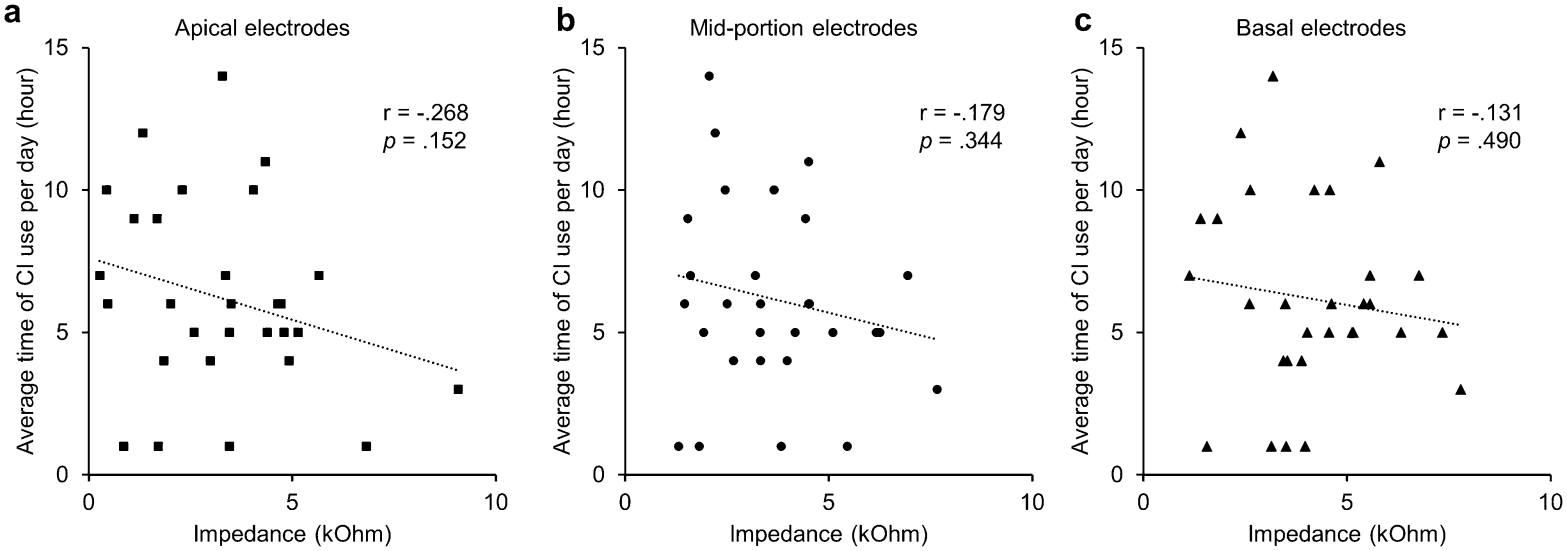
Correlation between device usage time and impedance change between the first and second mapping in the early switch-on group. The cochlear implant usage time does not correlate with changes in the measured impedance on the apical electrodes (**a**) as well as on the mid-portion (**b**) and basal electrodes (**c**).

We also investigated the changes in impedance according to the duration of CI device usage between the first and second mapping in the early switch-on group. Using Shapiro–Wilk normality test, we concluded that variables were normally distributed in 30 subjects who could provide the device usage time data; impedance changes in apical (W (30) = 0.957, *p* = 0.261), mid-portion (W (30) = 0.950, *p* = 0.164), basal electrodes (W (30) = 0.982, *p* = 0.873), and usage time (W (30) = 0.951, *p* = 0.179). Statistically, the Pearson correlation analysis was conducted, and the cochlear implant device usage time did not correlate to the average impedance change for all 22 electrode contacts (r = -0.209, *p* = 0.268), as well as the apical (r = -0.268 with *p* = 0.152), mid-portion (r = -0.179 with *p* = 0.344), and basal electrodes (r = -0.131 with *p* = 0.490). A scatterplot summarizes the results (Fig. 4).

## Discussion

One of the main findings of this study was that impedance measured within a period between 2 and 7 weeks after implantation of slim modiolar electrodes in the 26 subjects whose devices were activated on the day after surgery (early switch-on) were significantly lower than that in the subjects who underwent conventional switch-on between 2–3 weeks after surgery. The impedance values obtained intraoperatively were similar between the groups. However, the two groups showed interesting differences in the evolution of impedance according to the timing of initial fitting session. The average impedance level (MP2) in the conventional group increased by 32.7% (from 10.7 to 14.2 kΩ) in period 3 (2–4 weeks after surgery) compared to the intraoperative level, while that of the early switch-on group had decreased by 22.4% (from 11.5 to 8.9 kΩ). This increase in impedance level for the slim modiolar electrode array during the first 2–3 weeks before the initial switch-on in the conventional group was similar with that reported in previous studies with different types of electrode arrays, including the Nucleus 24 contour electrode array and the Nucleus standard straight array^21–23^. In contrast, other studies not using the slim modiolar electrode array, but using the similar early switch-on protocol, showed no significant decrease in impedance at 4 weeks after surgery compared to the values measured intraoperatively^18,20,24^. Saunders et al. and Hu et al. have indicated that different electrode array designs show different trends in longitudinal impedance changes over time^20,22^. To the best of our knowledge, this is the first study to report the time course of the postoperative impedance levels for a slim modiolar electrode array.

The second important finding of this study was that the significantly lower electrode contact impedance in the early switch-on group compared to the conventional switch-on group lasted nearly two months. In the conventional group, the impedance of all electrode contacts gradually increased after surgery and decreased with the use of electrical stimulation after device activation, and which was consistent with previous reports^7,21,22,25^. After implantation, the adsorption of protein onto the electrode surface and cell adherence to a protein layer are elicited as a foreign body immune response^26^. An in vitro study showed that the protein layer and cell growth increased electrode impedance^27^ and also revealed no significant stimulation-induced impedance changes in clean electrodes without cell coverage^28^. In addition, in both an in vivo study^7^ and a clinical study^25^, the reduction in impedance caused by stimulation was found to be larger when the impedance levels at 2 weeks after surgery were high. The effects of an inflammatory response on stimulation-induced changes in impedance have also been clinically studied. Tykocinski et al. reported an increase in polarization impedance up to 2 weeks after implantation caused by changes in the electrode–electrolyte interface, and a reversal of those impedance changes once electrical stimulation was initiated^6^. Previous studies have suggested that electrical stimulation may cause protein desorption and/or cell loss via electroporation over the electrode^7,27^, while it may have no effect on fibrous tissue growth around the electrode^6,9^. It is likely that the extent of the protein layer and cell growth in the early switch-on group continued to be lower than that in the conventional switch-on group for nearly 2 months. Thus, these findings indicate that early activation of the device partially prevented the increase in impedance associated with electrochemical reactions on the electrode surface. Empirically, providing low impedances in the early switch-on group at an early post-implant period was particularly beneficial for CI programming in a subgroup of patients who had abnormal findings on preoperative magnetic resonance imaging such as cochlear nerve deficiency (CND) or in elderly patients with a significantly smaller spiral ganglion neuron population. Indeed, in a bid to achieve auditory perception, CI recipients with CND required higher electrical charge per unit phase than those with normal anatomy^29^. In many cases, lowering impedance with the early switch-on protocol in such patients made it possible to reduce the number of out-of-compliance electrodes and allow for increased electric dynamic ranges from the early programming sessions.

Interestingly, the current study showed that impedance values stabilized much earlier in the early switch-on group than in the conventional switch-on group. For the patient group that underwent early switch-on, the measured values remained as low as 8.91 ± 1.66 kΩ (MP2) in period 3 (2–4 weeks after surgery) and stabilized thereafter. Conversely, in the conventional switch-on group, impedances decreased significantly from 14.21 ± 1.66 kΩ (MP2) in period 3 to 8.30 ± 1.55 kΩ (MP2) in period 6, and then remained relatively stable. Although the differences in impedance between the two groups were not significant for period 5 (*p* = 0.098), impedance remained slightly higher in the conventional switch-on group than in the early switch-on group (Fig. 2). Among the impedance components, the access resistance is generally determined by the electrolyte resistivity around the electrode and can be increased by the development of a fibrous tissue sheath. Since previous studies have indicated that the access resistance was not affected by electrical stimulation^7^, we speculated that changes in this impedance component over time may not be different between the two groups. Therefore, the lack of a significant difference in the impedance between the two groups in period 5 suggests that the reversible component, such as polarization impedance, in the conventional group was reduced after 2 months of electrical stimulation. As described above, assuming that early activation has the effect of preventing fluctuation of polarization impedance, when stabilization of the impedance had occurred as early as period 3, access resistance also did not increase 3–4 weeks after surgery. One possible factor that could lead to the early stabilization of access resistance is the electrode array type. The current study used a slim modiolar electrode array, which is designed to cause minimal intracochlear trauma during insertion and can be placed in a position close to the modiolus through a high degree of pre-curvation. It is reasonable to presume that less traumatic insertion and closer perimodiolar proximity may lead to the development of less fibrous tissue around the electrode, especially at the medial aspect of the electrode array, where the electrode contacts are facing and electrical stimulation does occur. We further analyzed whether the amount of time spent wearing the CI device was associated with a difference in impedance evolution within the population in the early switch-on group. It was found that when the initial activation was performed early, the frequency of CI device usage did not significantly affect the impedance evolution. Based on this consistent result of early stabilization of impedances using the slim modiolar electrode with the early switch-on protocol, a stable map was usually achieved 3–4 weeks after activation of the device. As a result, early switch-on of the CI device not only provided a nonstop rehabilitation program, but also decreased the number of hospital visits for CI fitting.

Previous studies have identified that the time course of impedance values differ according to the type of electrode array, and even within the electrode array the trend of impedance changes varies across the electrode array^18,20–22^. However, longitudinal changes in impedance over the long-term for slim modiolar electrode arrays have not been previously investigated. In the present study, electrode impedance values measured intraoperatively were highest for basal electrodes and lowest for apical electrodes. This could be due to the presence of air bubbles around the basal electrode contacts, which is related to the characteristic insertion technique of the slim modiolar electrode array^30^. Since this electrode array is loaded into the external polymer sheath before insertion, air trapped inside this sheath is commonly inserted into the cochlea with the electrode. Although the geometric surface area of the electrode contacts across the array gradually decreases in an apical direction (CI532, 0.19 mm2–0.16 mm2; CI632, 0.16 mm2–0.15 mm^2^), Fig. 3 shows no significant difference in impedance across the array about two weeks after surgery in both groups. It has been reported that impedance is inversely related to the geometric surface area of the electrodes^6^. This may be due to the combination of the two factors. First, it has been reported that there was more extensive fibrous tissue growth in the basal turn of the cochlea compared to the apical turn after implantation^9,25^. Second, the distance of the electrode in contact with the modiolar wall of the scala tympani was different. In this study, we used the round window insertion technique, in which the electrode was placed in a more medial position within the proximal basal turn by the crista fenestra at the inferior aspect of the round window compared to the electrode inserted via cochleostomy^31^. Although a favorable perimodiolar position of the electrode can be achieved by the slim modiolar electrode via the round window, a previous histological study has demonstrated that the distance of the electrode contact to the modiolar wall in the basal turn was more variable and farther apart than that in the apical and middle turns^32^. Thus, the impedance of the basal electrodes may be more vulnerable to the development of fibrous tissue because of its lateral placement.

As the safety and feasibility of the early activation of the CI device have been previously demonstrated, many centers have been encouraged to change their strategy to early switch-on. Also, no postoperative complications related to the early switch-on were observed in this study. However, other than the financial benefits of reducing the waiting period and the number of hospital visits^33^, the functional benefits of early switch-on have not yet been clearly reported. In this study, we found that at the initial switch-on, significantly lower impedance was associated with early switch-on of the slim modiolar electrode than the conventional switch-on, and the effect lasted nearly 2 months. We also observed that impedance stabilization was achieved as early as one month after surgery with an early switch-on strategy, which was 2 months earlier than that with the conventional switch-on strategy. Since sound quality and power consumption can be affected by impedance, stable impedances are essential for efficient and safe electrical stimulation. However, results from this study showed that no differences in the impedances between both groups were detected after 8 weeks implantation. While cochlear implants are designed to last a lifetime, the early switch-on of a CI is not likely to have a significant impact on impedance beyond that period. Moreover, we only investigated the impedance values in this study, which cannot lead to conclusions about the effects of the early switch-on strategy on clinical outcomes. Thus, further long-term research with psychophysical data, including thresholds, comfort levels, and dynamic ranges, will be needed to validate the functional benefits of early impedance stabilization.

The present study demonstrates that when applying the early switch-on strategy as early as 1 day after surgery for the slim modiolar electrode array, a much earlier stabilization of impedance can be achieved compared to the conventional switch-on strategy performed 2–4 weeks postoperatively. Additionally, the early switch-on strategy leads to comparatively lower impedance than the conventional switch-on strategy for at least 2 months postoperatively. Consequently, the early switch-on strategy has two advantages: (1) it may reduce hospital visits by one, and (2) it eliminates the stress of having to wait some weeks before knowing how the CI works.

## Supplementary Information

Supplementary Information.
