# Supplementary material for: Effect of initial switch-on within 24 hours of cochlear implantation using slim modiolar electrodes
Source: Sci Rep. 2021 Nov 23;11:22809. doi: 10.1038/s41598-021-01862-7 (PMC8611070; doi:10.1038/s41598-021-01862-7)
Supplement: Supplementary file 1 — Supplementary Information. [file 41598_2021_1862_MOESM1_ESM.pdf]

Effect of initial switch-on within 24 hours of cochlear implantation using slim modiolar electrodes

Woongsang Sunwoo[1, 3] · Hyoung Won Jeon[2, 3] · Byung Yoon Choi[2, *]

Supplementary Table S1. Mean impedance values of all channels of slim modiolar electrodes under CG mode.

	Period 1		Period 2	Period 3		Period 4		Period 5		Period 6		Period 7	
	**Conventional**	**Early**	**Early**	**Conventional**	**Early**	**Conventional**	**Early**	**Conventional**	**Early**	**Conventional**	**Early**	**Conventional**	**Early**
**Basal Electrodes (kΩ)**													
**CH 01**	10.74	12.09	4.19	12.74	7.43	9.88	7.82	9.48	7.63	9.04	8.18	9.14	8.39
**CH 02**	11.38	12.29	4.31	12.97	7.40	9.52	7.66	9.31	7.51	8.54	7.94	8.57	7.85
**CH 03**	11.79	12.25	4.30	13.24	7.94	9.65	7.88	9.36	8.00	8.44	8.19	8.56	7.95
**CH 04**	11.56	12.48	4.54	13.34	8.24	9.58	8.22	9.10	8.07	8.11	8.60	8.32	7.90
**CH 05**	11.42	12.54	4.55	13.10	8.01	9.45	8.29	8.91	7.85	7.83	8.03	8.03	7.78
**CH 06**	11.73	12.63	4.47	13.06	8.06	9.45	8.08	8.96	7.82	7.65	7.83	7.97	7.55
**CH 07**	11.43	12.06	4.46	12.75	8.05	9.39	8.11	8.90	7.77	7.50	8.17	7.89	7.45
**Mean (95% CI)**	11.44 (11.2-11.67)	12.33 (12.01-12.66)	4.4 (4.3-4.51)	13 (12.71-13.29)	7.94 (7.64-8.25)	9.56 (9.3-9.82)	8.01 (7.71-8.3)	9.15 (8.91-9.39)	7.81 (7.45-8.17)	8.16 (7.92-8.4)	8.13 (7.73-8.54)	8.35 (8.08-8.63)	7.84 (7.49-8.19)
**Mid-portion Electrodes (kΩ)**													
**CH 08**	10.62	11.51	4.36	12.56	7.91	9.29	8.24	8.64	7.83	7.31	8.09	7.67	7.52
**CH 09**	10.43	11.26	4.20	12.60	7.92	9.04	8.14	8.47	7.67	7.13	7.97	7.63	7.48
**CH 10**	10.19	11.00	4.21	12.53	8.05	8.97	8.11	8.48	7.68	7.10	7.52	7.55	7.46
**CH 11**	10.26	10.79	4.18	12.64	8.00	8.89	7.72	8.29	7.34	6.85	7.37	7.28	7.48
**CH 12**	10.23	10.54	4.33	12.52	7.98	8.86	7.94	8.14	7.28	6.72	7.71	7.24	7.35
**CH 13**	10.50	10.86	4.45	12.76	7.98	8.95	7.96	8.23	7.40	6.77	7.77	7.44	7.30
**CH 14**	10.49	11.34	4.59	12.98	8.41	9.12	8.02	8.38	7.76	6.88	7.86	7.56	7.95
**Mean (95% CI)**	10.39 (10.18-10.59)	11.04 (10.75-11.33)	4.33 (4.23-4.43)	12.74 (12.47-13)	8.14 (7.89-8.38)	9.02 (8.77-9.26)	8.02 (7.77-8.27)	8.37 (8.13-8.62)	7.57 (7.22-7.91)	6.96 (6.75-7.18)	7.76 (7.42-8.09)	7.48 (7.24-7.72)	7.51 (7.11-7.9)
**Apical Electrodes (kΩ)**													
**CH 15**	10.39	11.34	4.60	13.11	8.62	9.37	8.29	8.57	7.88	7.10	7.95	7.74	7.75
**CH 16**	9.94	11.04	4.51	13.13	8.71	9.23	8.45	8.48	7.78	7.17	8.22	7.59	7.52
**CH 17**	9.95	10.87	4.39	13.15	8.63	9.33	8.35	8.46	7.69	7.15	8.33	7.60	7.59
**CH 18**	10.02	11.19	4.56	13.21	8.82	9.53	8.55	8.56	7.77	7.32	8.13	7.51	7.78
**CH 19**	9.40	10.53	4.50	13.06	8.67	9.13	8.78	8.43	7.83	7.25	8.26	7.30	7.64
**CH 20**	9.11	10.21	4.51	12.95	8.60	9.27	8.59	8.47	7.41	7.15	7.89	7.62	7.17
**CH 21**	8.20	9.69	4.61	13.21	8.78	9.41	8.80	8.72	7.40	7.65	7.92	7.95	7.33
**CH 22**	9.31	10.00	4.49	13.41	8.77	9.60	8.06	8.65	7.27	7.53	7.44	7.62	6.78
**Mean (95% CI)**	9.54 (9.32-9.76)	10.61 (10.31-10.91)	4.52 (4.42-4.62)	13.11 (12.88-13.34)	8.59 (8.34-8.84)	9.36 (9.15-9.57)	8.48 (8.26-8.7)	8.54 (8.33-8.75)	7.63 (7.31-7.95)	7.29 (7.09-7.49)	8.02 (7.75-8.29)	7.62 (7.34-7.89)	7.45 (7.1-7.79)
**Total Electrodes (kΩ)**													
**Mean (95% CI)**	10.41 (9.91-10.91)	11.30 (10.59-12.00)	4.42 (4.22-4.63)	13.88 (13.46-14.29)	8.23 (7.58-8.87)	9.08 (8.53-9.63)	8.18 (7.59-8.77)	8.68 (8.10-9.27)	7.66 (6.83-8.50)	7.46 (6.96-7.97)	7.97 (7.20-8.75)	7.81 (7.14-8.47)	7.59 (6.78-8.40)
